# Vacuum aspiration for induced abortion could be safely and legally performed by nurses and midwives

**DOI:** 10.1136/jfprhc-2016-101542

**Published:** 2017-01-18

**Authors:** Sally Sheldon, Joanne Fletcher

**Affiliations:** 1 Professor of Law Kent Law School, Eliot College, Kent University, Canterbury, UK; 2 Consultant Nurse in Gynaecology, Sheffield Teaching Hospitals NHS Foundation Trust, Sheffield, UK

**Keywords:** abortion, family planning service provision, medico-legal

## Abstract

**Background:**

Some 40% of abortions carried out in England and Wales are done by vacuum aspiration. It is widely assumed that, in order to be lawful, these procedures must be performed by doctors.

**Aim and design:**

This study aimed to provide a detailed reassessment of the relevant law and the clinical evidence that supports this assumption.

**Conclusions:**

A close reading of relevant law reveals that this assumption is unfounded. On the contrary, it would be lawful for appropriately trained nurses or midwives, acting as part of a multidisciplinary team, to carry out vacuum aspiration procedures. This interpretation of the law offers the potential for developing more streamlined, cost-effective abortion services, which would be both safe and highly acceptable to patients.

## Introduction

It is widely assumed that, in order to be lawful when done for the purposes of inducing abortion, electric suction or manual vacuum aspiration procedures (VAs) must be performed by doctors. The attempt of Argent and Pavey, writing in this journal, to challenge this orthodoxy has gone unheeded.^1^ In its review of the Abortion Act (1967), the House of Commons Science and Technology Committee recognised the safety of allowing nurses and midwives to perform VAs but assumed that it would be unlawful for them to do so under existing law.^2^ Likewise, the Faculty of Sexual & Reproductive Healthcare (FSRH) of the Royal College of Obstetricians and Gynaecologists (RCOG) currently restricts training in surgical abortions to doctors, assuming that “[b]y current law, nurses and midwives are unable to perform surgical abortion procedures”, although they can “provide the medication prescribed by the doctor for medical abortions and assist in the provision of surgical procedures”.^3^ British abortion services are currently organised on the basis of this belief, with all surgical abortions in British clinics currently performed by medical doctors.

In this article, we argue that this orthodoxy is based on a flawed interpretation of relevant law. We suggest that, on the contrary, it would be lawful for appropriately trained nurses or midwives, acting as part of a multidisciplinary team, to carry out surgical abortions. Further, there is ample clinical evidence available to suggest that this would be perfectly safe. Indeed, there are already nurses working in the UK (including one of the current authors) who have demonstrated competence in performing VAs, where performed for miscarriage treatment or to remove retained products of conception (RPOC) post-miscarriage or abortion.

Recognising that appropriately trained nurses and midwives can safely and legally offer surgical abortions would better reflect existing legal precedent, provide a more appropriate recognition of nurse competences, follow government policy that patients should receive the right care, in the right place at the right time by appropriately trained staff,^4^ fit with guidance offered by relevant professional bodies, and offer the potential for developing more streamlined, cost-effective abortion services, which would be highly acceptable to patients. In what follows, we set out the relevant law before outlining the clinical evidence that supports our claim for the safety of permitting nurses and midwives to perform surgical abortions and briefly considering the implications of such a move.

## The law

“Unlawful procurement of miscarriage” is punishable by life imprisonment under the Offences Against the Person Act 1861. In 1967, the Abortion Act was introduced “to broaden the grounds upon which abortions may be lawfully obtained” and “to ensure that the abortion is carried out with all proper skill and in hygienic conditions”.^5^ The Act applies in England, Wales, Scotland but not Northern Ireland. It allows for abortions to be performed where two doctors certify in good faith that the woman meets one of a range of conditions; where the termination is performed by a registered medical practitioner; and where it is done on National Health Service (NHS) or other approved premises.

In this article, we focus on the second of these requirements, which aimed to ensure that abortions would be performed safely by an appropriately skilled professional. In 1967, the assumption that this would necessarily be a doctor reflected the medical fact that legal abortions were far riskier, technically more demanding procedures. In the words of one leading judge, they were “done by surgical methods”, with the “knife with the cutting edge” of necessity “operated by a registered medical practitioner”.^5^ In the years immediately following the introduction of the Act, however, the use of VA quickly became widespread in early pregnancy, already rendering the need for the skilled hand of a doctor less self-evident.

VA involves gentle suction to remove the fetus from the womb, typically taking less than 5 minutes. While it is used until approximately 15 weeks of pregnancy, the vast majority of procedures are done below 9 weeks.^6^ VA was introduced to much of the English-speaking world by a paper published in July 1967.^7^ By 1969, one-third of all induced abortions in England and Wales were performed this way.^8^ In 2015, 40% of abortions were done by VA, with a further 55% performed using abortion pills.^6^ Less than 1 in 20 abortions thus now rely on the more technically demanding methods that might clinically justify the need for the skilled hand of an experienced surgeon.

The courts considered the requirement that an abortion should be performed by a doctor in *RCN* v *DHSS* [1981],^5^ a case involving second-trimester medical terminations. At the time, a doctor's involvement in performing this kind of abortion was typically limited to the insertion of a catheter into the woman's womb. After that, while available to be called if necessary, he or she would not routinely be present on the ward for the 18–30 hours that it could take for the abortion to occur.^5^ Rather, it would be nurses or midwives who would attach the catheter to a pump, add the necessary infusion of prostaglandins, turn on the pump, monitor the patient's vital signs and adjust the flow of prostaglandins as necessary.

In *RCN*, the courts were asked to consider the following question: where the only steps that directly cause an abortion are carried out by a nurse or midwife, is the pregnancy “terminated by a registered medical practitioner”? By a slim majority, three of the five judges who heard the case in the House of Lords (then the highest UK appellate court) found that it was: this provision required that a doctor “should accept responsibility” for all stages of treatment for the termination of pregnancy, without necessarily needing to carry out specific actions him or herself.^5^ Lord Diplock explained:

*“The particular method to be used should be decided by the doctor in charge of the treatment for termination of the pregnancy; he should carry out any physical acts, forming part of the treatment, that in accordance with accepted medical practice are done only by qualified medical practitioners, and should give specific instructions as to the carrying out of such parts of the treatment as in accordance with accepted medical practice are carried out by nurses or other members of the hospital staff without medical qualifications. To each of them, the doctor, or his substitute, should be available to be consulted or called on for assistance from beginning to end of the treatment.”* 5

This reading allowed the existing medical practice for second-trimester medical inductions to be maintained. It is also taken as the basis of the legality of the now common practice of allowing an appropriately trained nurse to hand over or to administer abortion pills that have been prescribed by a medical practitioner.

How does this apply to surgical abortions? It is widely assumed that, in order to be legal, these must be performed by doctors. However, this assumption is difficult to square with the decision in *RCN*. Rather, subject always to the clinical safety of such a move, the better understanding is that surgical abortions might be legally performed by an appropriately trained and skilled nurse or midwife, acting as part of a multi-disciplinary team that includes a doctor. In such a case, the doctor's role in deciding upon treatment and giving any necessary, specific instructions as to how it should be carried out would be exactly the same. Further, he or she (or a substitute) would be available to be consulted or called upon for assistance throughout the treatment. While it might appear to stretch the statutory language to interpret medical direction and oversight as sufficient to constitute ‘performance’ of a hands-on, surgical procedure, it should be recalled that, on its facts, *RCN* was also concerned with the performance of physical acts.

Our interpretation of the law is supported by a careful reading of the three majority judgments offered in the House of Lords in *RCN*. Lord Keith finds that a doctor must have “responsibility for the whole process” and “personally [perform] essential parts of it which are such as to necessitate the application of his particular skill”. This formulation implicitly accepts that the question of what tasks must be performed by a doctor should be resolved with reference to evidence of best clinical practice.^5^ Lord Roskill reasons that the legal requirement is met when the “entirety of the treatment for the termination of pregnancy and [the nurse's] participation in it is at all times under the control of the doctor even though the doctor is not present throughout the entirety of the treatment”.^5^ Likewise, Lord Diplock finds that “the doctor need not do everything with his own hands”. Rather, treatment should be “carried out in accordance with his directions”, with a doctor remaining “in charge throughout”.^5^ Each of the three majority judgments thus offers strong support for the view that it is legal for appropriately trained nurses and midwives, acting as part of a multidisciplinary team that includes a doctor, to perform surgical abortions.

Our interpretation of the law is further supported by recent discussion in the Supreme Court (which, in 2009, replaced the Appellate Committee of the House of Lords as the highest court in the UK). Citing *RCN,* the Court's Deputy President, Lady Hale, noted that the statutory requirement was met:

*“… when* [the abortion] *was a team effort carried out under* [the doctor's] *direction, with the doctor performing those tasks that are reserved to a doctor and the nurses and others carrying out those tasks which they are qualified to perform”.* 9

The Court thus did not read the Abortion Act as requiring that a doctor perform any specific physical tasks. Rather, “reserved to a doctor” might be understood as referring to those aspects of treatment where other regulation requires that a doctor perform them, such as certification requirements or the right to prescribe certain medicines (including the mifepristone and misoprostol used in a medical abortion). Alternatively, the implied opposition between “reserved to a doctor” and those tasks which others are “qualified to perform” might be taken to imply that the former is appropriately understood as meaning those tasks that a doctor alone is qualified to perform. As we show next, a review of the clinical evidence clearly shows that this would not apply to vacuum aspiration.

## The clinical evidence

The World Health Organization recommends that nurses and midwives can be trained to provide safe, early abortion without compromising safety.^10^ ^11^ This recommendation reflects the clinical evidence, which shows no difference in complication rates between women who had first-trimester abortions with VA performed by mid-level healthcare providers (nurses, midwives and other non-physician providers) and those who had the procedure performed by a doctor. One systematic review of five studies from the USA, Nepal, South Africa, Vietnam and India, including two randomised controlled trials and three prospective cohort studies, compared the experience of 4198 women who underwent a procedure administered by a mid-level provider, and 4341 who underwent a physician-administered procedure.^12^ While complications were rare amongst both provider groups, the review revealed no statistical differences in incomplete abortion and complications for first-trimester surgical and medical abortion up to 9 weeks performed by mid-level providers compared with doctors. A further prospective, observational study, which evaluated the outcomes of 5812 VAs performed by doctors and 5675 newly trained nurse practitioners, midwives and physician assistants in California, equally found no difference in complication rates.^13^

Turning specifically to the UK, while in 1967 it was uncommon for nurses or midwives to perform surgical procedures, today they perform a range of complex procedures including colposcopies and hysteroscopies.^1^ They also fit intrauterine devices and intrauterine systems, a procedure that is said to require about the same level of skill as VA.^2^ In its review of the scientific evidence relating to abortion, the Science and Technology Committee of the House of Commons recommended that, subject to usual training and professional standards, nurses and midwives should be permitted to carry out early surgical abortions, finding that this would not compromise patient safety or quality of care.^2^

Moreover, nurses in Britain are already performing VAs under local anaesthetic for surgical management of miscarriage or RPOC post-miscarriage or abortion. Whilst the numbers of nurses and midwives in England performing VAs are low, one of the authors and two of her nursing colleagues have been providing a manual VA service since 2011. The nurse-led service was established in order to improve the patient experience, avoiding the situation where women might sometimes wait days for access to a surgical procedure that was historically reliant on the availability of a doctor. In the absence of any formal accredited competency-based training programme for nurses and midwives, a local training package was devised based on the FSRH VA for abortion training module.^3^ The service is now very much reactive to the needs of women (miscarriages and emergencies cannot be predicted), has streamlined surgical management for women experiencing miscarriage or RPOC, and has improved the patient experience, with women no longer waiting for a slot on an emergency theatre list. Usually women can arrange a procedure on a date and time to suit their circumstances, knowing that they will only be in hospital for a few hours.

An internal audit of the first 50 cases performed by the nursing staff showed that only one patient required further oral misoprostol for continued bleeding post-procedure. While the service has generally proved a great success, it has led to some confusion: women who may have had a VA performed by a nurse following a miscarriage do not understand why they cannot opt for a nurse to perform the same procedure for their requested abortion, particularly when this creates delay in their ability to access a legally authorised procedure.

Development of nurse-led surgical abortion services would be in line with the development of nursing competences and the four dimensions of the Nursing and Midwifery Council *The Code* (2015).^14^ This requires prioritising people, practising effectively, preserving safety, and promoting professionalism and trust. In line with these core values, the introduction of a nurse-led surgical abortion would recognise the needs of women requesting abortion, assess and respond to them in a timely manner, and offer a choice of procedure by appropriately trained, competent staff, potentially at an earlier gestation than where necessary to wait for a place on a surgical list offered by a doctor. The RCOG has already noted its explicit support for according greater responsibility to nurses involved in abortion service provision.^15^

Finally, at a time of extreme budgetary restraint within the NHS, the current understanding of the law has resulted in the (relatively expensive) time of doctors being devoted to work that might safely be done by nurses and midwives. The latter are also likely to constitute a more stable workforce than junior doctors, who will move around during their career progression. Allowing nurses to provide this service thus potentially offers a more sustainable and economically efficient basis for the long-term development of excellent care. It would free up doctors to focus on those aspects of service provision where their specific expertise is needed. It might also improve the job satisfaction of nursing and midwifery staff, potentially impacting positively on sickness absence and staff retention rates.

Our interpretation of the law thus offers the potential for improved services, whilst also better reflecting existing legal precedent. While we are confident that our reading of the law is the correct one, given that the alternative, current reading of the law is so well entrenched, it may be advisable to seek judicial clarification before any reorganisation of services takes place. This could be done by seeking a court declaration. The FSRH might then open its training modules in surgical abortion to nurses and midwives, allowing them to develop the competences that would allow them to perform procedures safely and effectively. As with any innovation in clinical practice, the broadening of the range of professionals who are permitted to perform VAs should be closely monitored and reviewed. However, the available clinical evidence gives every ground for strong confidence that such monitoring will confirm its safety.

## Conclusions

Our reading of the relevant law stands in clear contrast to that which has informed the current organisation of abortion services. However, we do nothing more than to take the well-established interpretation of the relevant provision of the Abortion Act offered by our highest domestic court to its natural conclusion. Moreover, our interpretation of the law adheres closely to the broad purposes of the Abortion Act, as laid down in *RCN*.^5^ The recognition that, subject to appropriate training, it would be lawful for nurses and midwives to offer VAs would have no effect on the Act's first purpose (to extend the grounds upon which abortions can be provided) and it should be permitted only if it conforms to the second (to ensure that the abortions are carried out with all proper skill and in hygienic conditions). Indeed, it might be suggested that once a termination has been duly authorised, it is only the second of these purposes that is relevant. In conclusion, if VAs can be safely carried out by appropriately trained and skilled mid-level providers, there is no reason not to accommodate this practice within the existing statutory framework.
